# Letters to the Editor: Indeterminate form of Chagas Disease: some immunological insights

**DOI:** 10.1590/0037-8682-0713-2021

**Published:** 2022-04-08

**Authors:** Alejandro Marcel Hasslocher-Moreno, Sergio Salles Xavier, Roberto Magalhães Saraiva, Andréa Silvestre de Sousa

**Affiliations:** 1 Fundação Oswaldo Cruz, Instituto Nacional de Infectologia Evandro Chagas, Rio de Janeiro, RJ, Brasil.; 2 Universidade Federal do Rio de Janeiro, Faculdade de Medicina, Rio de Janeiro, RJ, Brasil.

We appreciate the letter to the editor of Matos et al.. The authors present their experience in evaluating the role of CD14+/HLA-DRlow/‒ monocytes and CD4+ and CD8+ T lymphocytes in various clinical forms of chronic Chagas disease^1^.

First, I would like to emphasize that patients with asymptomatic Chagas disease do not necessarily have the indeterminate form, as there are asymptomatic patients among those in the initial stages of the cardiac form. Therefore, we prefer to use the term clinical progression to identify the transition from indeterminate form to cardiac form^2^.

When evaluating the clinical progression of patients in the indeterminate form, it is important to consider whether they were previously treated with trypanocidal drugs. There is a significant difference in progression rates from indeterminate form to cardiac form between treated and untreated patients^3^. All patients included in the data presented by Matos et al. had a history of previous treatment with benznidazole, which can improve the functional capacity of CD8+ and CD4+ T cells^4^ and influence their results of similar quantification of CD4+/CD8+ T lymphocytes across different forms of Chagas disease.

Another relevant aspect of this discussion is the fact that only through prospective studies, where long-term follow-up of individuals is carried out, it is possible to correlate the findings with the clinical forms of the disease^5^ ^,^ ^6^. Cross-sectional studies do not allow us to infer cause-effect relationships, but plausible hypotheses may be raised. Few studies have evaluated the prognostic value of biomarkers over time, including but not limited to BNP, transforming growth factor β1, and metalloproteinase^7^ ^-^ ^9^. To the best of our knowledge, no study has evaluated the long-term prognostic value of the quantification of CD4+/CD8+ T lymphocytes. With only long-term follow-up studies, we will be able to infer a role for innate immunity, represented by macrophages and neutrophils, dendritic cells, and natural killer lymphocytes; and for adaptive immunity by B lymphocytes and T lymphocytes^10^ in the clinical progression of the cardiac form of the disease.
